# SPOT-Disorder2: Improved Protein Intrinsic Disorder Prediction by Ensembled Deep Learning

**DOI:** 10.1016/j.gpb.2019.01.004

**Published:** 2020-03-13

**Authors:** Jack Hanson, Kuldip K. Paliwal, Thomas Litfin, Yaoqi Zhou

**Affiliations:** 1 Signal Processing Laboratory, Griffith University, Brisbane 4111, Australia; 2 School of Information and Communication Technology, Griffith University, Gold Coast 4222, Australia; 3 Institute for Glycomics, Griffith University, Gold Coast 4222, Australia

**Keywords:** Intrinsic disorder, Molecular recognition feature, Machine learning, Deep learning, Protein structure

## Abstract

Intrinsically disordered or unstructured proteins (or regions in proteins) have been found to be important in a wide range of biological functions and implicated in many diseases. Due to the high cost and low efficiency of experimental determination of **intrinsic disorder** and the exponential increase of unannotated protein sequences, developing complementary computational prediction methods has been an active area of research for several decades. Here, we employed an ensemble of deep Squeeze-and-Excitation residual inception and long short-term memory (LSTM) networks for predicting protein intrinsic disorder with input from evolutionary information and predicted one-dimensional structural properties. The method, called SPOT-Disorder2, offers substantial and consistent improvement not only over our previous technique based on LSTM networks alone, but also over other state-of-the-art techniques in three independent tests with different ratios of disordered to ordered amino acid residues, and for sequences with either rich or limited evolutionary information. More importantly, semi-disordered regions predicted in SPOT-Disorder2 are more accurate in identifying **molecular recognition features** (MoRFs) than methods directly designed for MoRFs prediction. SPOT-Disorder2 is available as a web server and as a standalone program at https://sparks-lab.org/server/spot-disorder2/.

## Introduction

Intrinsic disorder in proteins is the lack of tendency of a protein to fold into a well-defined, rigid structure. These dynamic protein structures can be experimentally observed as their backbone angles vary over time due to their innate flexibility [1]. The discovery of intrinsically disordered proteins (IDPs) or intrinsically disordered regions (IDRs) in proteins challenged the dogmatic structure–function paradigm, forcing a new perspective where protein rigidity is no longer a foregone conclusion [2].

IDPs are able to fulfil a wide range of niche, yet biologically crucial functional roles despite their lack of a rigid structure, due to their ability to transition between a set of transient, interconverting structural states [3]. Advantaged by their disordered flexibility [4], IDPs play essential roles in signaling, assembling, and regulatory functions [5], and are implicated in numerous human diseases, such as cancer, amyloidoses, cardiovascular disease, neurodegenerative diseases, and various genetic diseases [6]. A recent study on the amino acid (AA) residue-wise coverage of disorder has estimated that 19.6% of AA residues in eukaryotic organisms and 9.6% of AA residues in viral organisms are disordered [7]. This prevalence is vindicated by the fact that naturally-occurring proteins, particularly those in eukaryotes and viruses [7], [8], [9], tend to be more disordered than random sequences [10]. Thus, determining the identity and locations of IDPs and IDRs is fundamental to understanding and addressing the cause and effect of these unstructured states [11].

Due to the extensive monetary and time cost of experimental procedures, such as nuclear magnetic resonance (NMR), X-ray crystallography, and circular dichroism (CD) [12], [13], many computational methods have been designed to bridge the growing gap between unannotated and annotated protein structures and/or their intrinsic disorder. Early work in protein disorder prediction was often based on small machine learning models [14], such as neural networks and support vector machines (SVMs). Other computational methods calculated disorder through the analysis of AA propensities and other sequence properties [15]. As more data and powerful tools became available, deep learning and recurrent architectures have taken the forefront, in methods such as SPINE-D [16], ESpritz [17], AUCpreD [18], SPOT-Disorder [19], and NetSurfP-2.0 [20]. A recent review by Liu et al. [21] placed SPOT-Disorder and AUCpreD as the two top-performing predictors for protein disorder prediction.

SPOT-Disorder [19], previously introduced by us, employed long short-term memory (LSTM) cells [22] in a bidirectional recurrent neural network (BRNN) [23] for protein disorder prediction. Since the publication of SPOT-Disorder, the single LSTM-BRNN topology for deep learning has been enhanced by utilizing an ensemble set of hybrid models consisting of both LSTM-BRNNs and residual convolutional neural networks (residual CNNs, called ResNets) [24] for protein contact map prediction [25], protein ω angle prediction [26], and protein secondary structure prediction [27]. This architecture of network ensembles is advantageous because it can congregate and propagate both short-distance (ResNets) and long-distance (LSTM-BRNN) interactions throughout the protein sequence. Furthermore, the residual connections in these models alleviate the issues brought about by the vanishing gradient problem and allow for much deeper models (in the case of CNNs) and more effective gradient flow.

The effectiveness of ResNets and their various derivatives is displayed by their high performance in recent image classification competitions (ImageNet) [28]. Two such derivatives yet to be applied in bioinformatics are residual-inception networks [28] and Squeeze-and-Excitation networks [29]. Inception networks (v4) expand on the basic ResNets by increasing the number of paths available for data to be passed through. As such, the identity mapping function provided by the residual connection has a deeper level of abstraction due to the independent data paths. Squeeze-and-Excitation networks are another effective modification to ResNets that compresses the passing information into an excitation signal. This excitation signal can control the specific values added to the residual connection through the convolutional paths, behaving similarly to the learned gates of an LSTM cell. These models are currently cutting edge in image and speech processing tasks.

In this work, we examine models incorporating inception paths, residual connections, and Squeeze-and-Excitation networks (IncReSeNet) for their usefulness in disorder prediction. We find that the ensemble of different deep learning models leads to a stable and superior performance in four independent test sets with different ratios of ordered to disordered AA residues.

## Methods

### Neural network

The neural network topology employed in SPOT-Disorder2 consists of various models sequentially combining IncReSeNet, LSTM, and fully-connected (FC) topographical segments. Several models have been individually trained and then combined as an ensemble by averaging the disorder prediction output from each model. The hyperparameters of each individual method are outlined in Table 1.

**Table 1:** **Architecture of five network models in the ensemble**

**Model**	**First segment**	**N_LSTM_**	**N_CNN_**	**K_CNN_**	**N_FC_**	**No. of blocks**		
						**LSTM**	**CNN**	**FC**
0	RNN	250	60	5	250	2	10	1
1	RNN	250	60	7	500	2	10	1
2	RNN	250	60	9	250	2	10	1
3	CNN	250	60	9	250	2	10	2
4	RNN	250	60	9	250	1	10	1

*Note*: The order of layers in the model is presented in the column for the first segment, with LSTM and IncReSeNet blocks being the first segment for RNN and CNN, respectively (*i.e.*, at the input layers). N_LSTM_, N_CNN_, and N_FC_ refer to the number of hidden units in each LSTM cell, convolutional filters in each CNN layer, and nodes in each FC block, respectively. K_CNN_ refer to the kernel size of CNNs. LSTM, long short-term memory; RNN, recurrent neural network; CNN, convolutional neural network; FC, fully-connected layer; IncReSeNet, model incorporating inception paths, residual connections, and Squeeze-and-Excitation networks.

**Figure 1:**
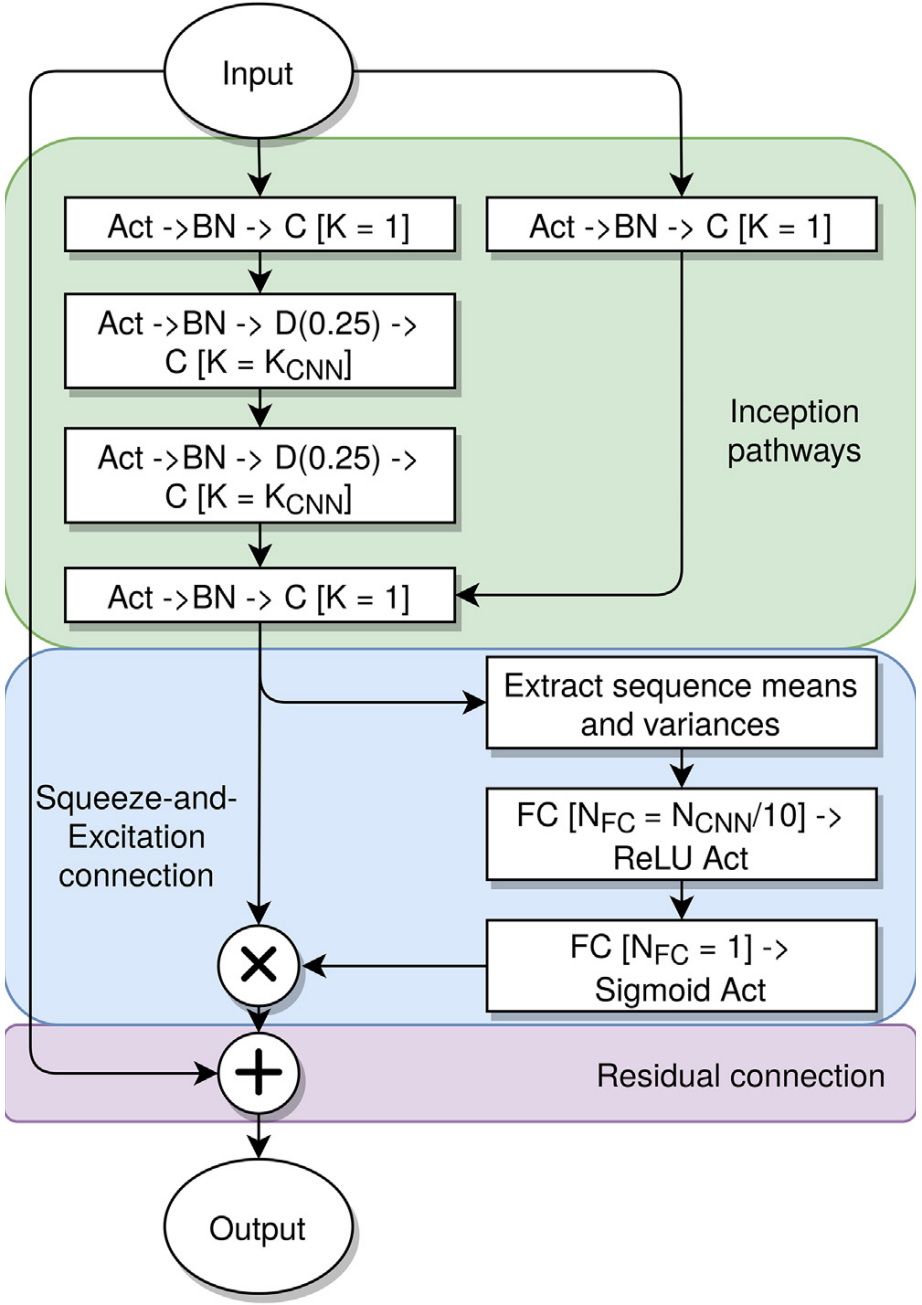
**IncReSeNet blocks** This plot shows the data pathways from the input (top) to the output (bottom) of each IncReSeNet block. The Squeeze-and-Excitation (blue) section takes the output of the inception paths (green) and uses this information to control how much of itself is output from this block onto the residual pathway (purple). This is repeated for each sequential IncReSeNet block. The network-dependent parameters are detailed in Table 1. IncReSeNet, model incorporating inception paths, residual connections, and Squeeze-and-Excitation networks; BN, batch normalization; Act, activation; C, 1D convolution with kernel width K_CNN_; D(0.25), dropout of 25%; FC, fully-connected layer; K, parameter denoting layer kernel size; CNN, convolutional neural network; N_FC_, number of neurons in FC; N_CNN_, number of nodes in each convolutional layer; ReLU, rectified linear unit.

The IncReSeNet segments follow the order of operations in the pre-activation ResNets architecture [24], with a multi-path inception-style architecture similar to Inception V4 [28]. As shown in the flow diagram (Figure 1), each block has three branches, including the residual connection and two convolution paths with 3 and 1 convolution operation, respectively. Each convolution operation is performed with a one-dimensional (1D) kernel with its size denoted as K_CNN_, except for the first convolution in each branch, which has a kernel size of 1. These two paths are then concatenated and passed into a convolution of kernel size 1 and its depth denoted as N_CNN_. The input to every convolution is normalized by the batch normalization technique [30], and is then activated by the exponential linear unit (ELU) activation function [31]. As each residual connection is preactivated, at the conclusion of all of the IncReSeNet layers, the output is both normalized and activated. Dropout of 25% is applied internally in some of the InReSeNet convolutions to avoid overtraining [32]. As shown in Figure 1, dropout is applied after batch normalization (to not affect the moving average and variance measurements), but before the convolution operations (to not to affect the residual connection).

The Squeeze-and-Excitation segments in the residual blocks consist of two FC layers applied directly before the residual connection is applied. The means and variances across the protein for the outputs of the prior convolution layer are calculated to provide 2 × N_CNN_ values per protein. These values are then passed through two FC layers with N_CNN_/10 outputs and a single output, respectively, and an ReLU and sigmoid activation. The outputs of the second FC layer are then used as a makeshift logic gate to select which values from the final convolution of the block will be added to the residual connection.

The LSTM layers follow a similar format to our previous experiments [19], [27]. Each LSTM block consists of one bidirectional LSTM layer with a memory cell size annotated as N_LSTM_ in both directions, resulting in N_LSTM_ × 2 output values. Dropout of 50% is applied to the output of the LSTM blocks. Each FC layer’s size is denoted as N_FC_, is activated by a rectified linear unit (ReLU) [33] and regularized by dropout. No dropout is employed for the output layer, which employs a sigmoid activation to convert the singular output into a probability of the AA residue being disordered.

The use of an ensemble predictor minimizes the effect of generalization errors between models [34]. A large corpus of models with varying hyperparameters are trained and their performance is analyzed on a validation set. These hyperparameters are swept through in a grid search and include the layout of the network, the number of nodes in each layer (one parameter each for LSTM, IncReSeNet, and FC layers), and the number of layers for each layer type. The five top-performing models with hyperparameters listed in Table 1 are chosen from this validation period and used in the final ensemble for SPOT-Disorder2. Selecting more models did not contribute to an increase in accuracy (data not shown).

SPOT-Disorder2 has been trained using the inbuilt Adam optimizer [35] in TensorFlow v1.10 [36], on an NVIDIA TITAN X GPU. A typical IncReSeNet model takes 40 s/epoch over our whole training set, whereas an LSTM network takes 3 min/epoch.

### Input features

SPOT-Disorder2 employed a similar set of features to SPOT-Disorder. Besides the same evolutionary content consisting of the position-specific substitution matrix (PSSM) profile from PSI-BLAST [37], SPOT-Disorder2 also includes the hidden Markov model (HMM) profile from HHblits [38]. The PSSM profile is generated by 3 iterations of PSI-BLAST against the UniRef90 sequence database (UniProt release 2018_03), and consists of 20 substitution values of each position for each AA residue type. The HMM profile consists of 30 values generated by using HHblits v3.0.3 with the UniProt sequence profile database from Oct 2017 [39]. These 30 values themselves consist of 20 AA substitution probabilities, 10 transition frequencies, and the number of effective homologous sequences of a given protein (Neff). In addition, we utilized the predicted structural properties from SPOT-1D [27], a significant update from SPOT-Disorder which utilized SPIDER2 [40], [41]. The features from SPOT-1D consist of 11 secondary structure probabilities (both three- and eight-state predicted secondary structure elements), 4 sine and 4 cosine θ, τ, φ, and ψ backbone angles, 1 relative solvent-accessible surface area (ASA), 1 contact number (CN), and 2 half-sphere exposure (HSE) values based on the carbon-α atoms.

These feature groups are concatenated to form 73 input features for each protein residue. Features of each residue are standardized to have zero mean and unit variance before being input in the network by the means and standard deviations of the training data.

### Datasets

The datasets used in these experiments, as shown in Table 2, were obtained from our previous disorder prediction publications [16], [19]. To summarize, we obtained 4229 non-redundant, high-resolution protein sequences from the Protein Data Bank (PDB) and Database of Protein Disorder (DisProt) [42]. These include 4157 X-ray crystallography structures (deposited to the PDB prior to August 05, 2003) and 72 fully-disordered proteins from DisProt v5.0. These chains were randomly split into a training set (Training) of 2700 chains, a validation set (Validation) of 300 chains, and a testing set (Test) of 1229 chains. Sequence similarity among these proteins is <25% according to BLASTClust [37]. As SPOT-1D has not been trained for proteins of length >700 AA residues, we remove all proteins of length >700 from all datasets. This reduces our training, validation, and test sets to 2615, 293, and 1185 proteins, respectively. For convenience, we will label this test set as Test1185.

**Table 2:** **Order and disorder propensity in each of the datasets**

**Dataset**	**No. of proteins**	**No. of ordered residues**	**No. of disordered residues**	**Percentage of disorder**
Training	2615	542,532	59,743	9.92%
Validation	293	57,470	5765	9.12%
Test1185	1185	246,616	26,515	9.71%
SL250	250	32,261	21,173	39.62%
Mobi9414	9414	1,932,536	127,362	6.18%
DisProt228	228	30,772	18,811	37.94%

We also obtained three independent test datasets (SL250, Mobi9414, and DisProt228) for a fair comparison against other methods. These datasets were the subsets from the established SL477 [16], MobiDB [43], and DisProt Complement [44] sets, respectively, after removing long proteins (>700 residues) and homologous proteins in our training dataset (25% sequence identity cutoff with BLASTClust). The proteins in DisProt228 are newly-annotated proteins that are deposited in the DisProt database v7.0 [45]. The proteins in SL477 with unknown residue types were also removed. The annotations in Mobi9414 (*i.e.*, from MobiDB) contain direct labels from the DisProt database, inferred labels from the PDB, and predicted labels from a large ensemble of disorder predictors such as ESpritz [17]. Predicted labels in MobiDB are not utilized due to their potential inaccuracy. Residues listed as ‘conflicting’ labels in MobiDB are omitted for performance analysis. Because some predictors employed MobiDB as a part of their training set, we also made a reduced subset of the Mobi9414, called Mobi4730 for independent testing for all methods compared. Because not all training sets are available for all methods, Mobi4730 was obtained by clustering Mobi9414 against the largest disorder training dataset for NetSurfP-2.0 at a sequence similarity of 25% by BLASTClust.

### Performance evaluation

Analyzing the performance of a disorder predictor is difficult due to the innate class imbalance present between disordered and ordered AA residues. As such, several skew-independent metrics are used to gauge the overall classification accuracy of the predictor. They include sensitivity (the fraction of predicted positives in all true positives), precision (the fraction of true positives in predicted positives), specificity (the fraction of true negatives in predicted negatives), the weighted score Sw (Sw = sensitivity + specificity − 1), the area under the receiver operating characteristic (ROC) curve (AUC_ROC_), and the area under the precision–recall curve (AUC_PR_). The difference between two AUC_ROC_ values can be qualified as statistically significant according to a *P* value from a bivariate statistical test [46], where a smaller *P* value indicates a higher likelihood of the difference being significant. AUC_PR_ emphasizes the performance on positive labels, which is particularly informative when the fraction of positive labels is low, as the case of protein disorder [47].

In addition, we obtain the Matthew’s correlation coefficient (MCC) between the predicted and true labels with $\mathrm{MCC}=\left(TP\times TN+FN\times FP\right)/\sqrt{\left(TP+FP)(TP+FN)(TN+FP)(TN+FN\right)}$, where TP, TN, FP, and FN indicate true positive, true negative, false positive, and true negative, respectively.

These metrics all have a maximum value of 1, and as such the highest performing predictor can be taken as the one that provides the overall highest metrics across our testing datasets.

### Method comparison

We compare SPOT-Disorder2 to several high-performing protein disorder predictors. These include the local versions of DISOPRED2 [48] and DISOPRED3 [49] (http://bioinfadmin.cs.ucl.ac.uk/downloads/DISOPRED/), MobiDB-lite [50], AUCpreD [18] (https://github.com/realbigws/RaptorX_Property_Fast), s2D [51] (http://www-mvsoftware.ch.cam.ac.uk/index.php/s2D), SPOT-Disorder [19], SPOT-Disorder-Single (denoted as SPOT-Disorder-S for brevity) [52], and SPINE-D [16] (http://sparks-lab.org/). We also used the webserver of NetSurfP-2.0 [20] (http://www.cbs.dtu.dk/services/NetSurfP-2.0/). Additionally, different versions of ESpritz method [17] were downloaded (http://protein.bio.unipd.it/download/), which were based on either single-sequence (seq) or sequence profile (prof) information. These ESpritz methods were trained based on structural information obtained from DisProt database, or PDB as determined by NMR or X-ray crystallography, which were termed as ESpritz-D, ESpritz-N, and ESpritz-X, respectively.

### Application to prediction of molecularSPOT-Disorder2 were obtained by Necci recognition motifs

In order to predict molecular recognition features (MoRFs), we define two thresholds as upper and lower bounds to classify the outputs of SPOT-Disorder2 as MoRFs. We also smooth the outputs of SPOT-Disorder2 to prevent the prediction of short MoRFs regions of <3 residues, since MoRFs typically are longer based on our analysis. To do this, we apply a sliding window of size *w_L_* to the predicted labels (*y_M_*) of SPOT-Disorder2, and apply the following function $\hat{y_{m}}\left(i\right)=1\;if\sum y_{m}\left(j\right)>w_{L}$, and 0, if otherwise.

## Results and discussion

### Importance of ensembled learning and features for disorder prediction

One novel aspect of SPOT-Disorder2 is the use of an ensemble of IncReSeNet, LSTM, and FC network topologies, rather than a single LSTM topology in the previous version (SPOT-Disorder). Thus, it is necessary to examine if additional network models lead to an improvement of SPOT-Disorder2 over SPOT-Disorder. As shown in Table 3**,** there is a clear significant improvement across all test datasets based on four different measures (AUC_ROC_, AUC_PR_, MCC, and Sw). For example, MCC values are improved by 7%, 8%, 7%, and 8% for the four independent test sets of Test1185, SL250, Mobi9414, and DisProt228, respectively. Improvement on AUC_PR_ is less consistent with 7%, 2%, 13%, and 8% improvement for the four test sets, respectively, because AUC_PR_ is very sensitive to precision at low sensitivity.

**Table 3:** **Performance of the SPOT-Disorder2 and SPOT-Disorder on four independent test datasets**

**Dataset**	**SPOT-Disorder2**				**SPOT-Disorder**			
	**AUC_ROC_**	**AUC_PR_**	**MCC**	**Sw**	**AUC_ROC_**	**AUC_PR_**	**MCC**	**Sw**
Validation	0.938	0.725	0.621	0.739	–	–	–	–
Test1185	0.914	0.698	0.607	0.676	0.894	0.65	0.567	0.477
SL250	0.901	0.889	0.679	0.625	0.893	0.875	0.629	0.567
Mobi9414	0.943	0.71	0.642	0.744	0.924	0.628	0.598	0.567
DisProt228	0.81	0.722	0.5	0.452	0.793	0.668	0.463	0.465

*Note*: For SPOT-Disorder2, both MCC and Sw are obtained using thresholds that maximize MCC and Sw on the Validation set, (thresholds are 0.370 for MCC and 0.070 for Sw, respectively). AUC_ROC_, area under the receiver operating characteristic curve; AUC_PR_, area under the precision–recall curve; MCC, Matthew’s correlation coefficient; Sw, weighted score.

To demonstrate the effectiveness of using an ensemble over using a single model for intrinsic disorder prediction, we compared the performance of the single component models to that of the ensemble using the Mobi9414 dataset. As shown in Table S1, the use of ensembled learning enables more accurate final output when compared to the Model 2, the highest-performing component model on this dataset. However, Model 4, rather than Model 2, is the highest performing component for Test1185 (MCC of 0.599 and 0.593 for Models 4 and 2, respectively, against 0.607 for SPOT-Disorder2). This variation in model ranking for different test sets indicates the effectiveness of ensembling in increasing the robustness of the culminating model.

To examine the contribution of each feature type to the performance of SPOT-Disorder2, we analyzed the performance of Model 0 for the Mobi9414 dataset. The features have been separated into groups provided by the following programs: PSI-BLAST (PSSM), HHblits (HHblits), and SPOT-1D (SPOT-1D). As shown in Table S2, PSSM is the most critical feature for maximizing both AUC metrics, while SPOT-1D is the most critical for enhancing the single threshold metrics MCC and Sw. HHblits, on the other hand, does not seem to have a significant contribution to the performance of the model, probably because the HHblits profile has already been used in the input pipeline through SPOT-1D. The difference between AUC_ROC_ for the full Model 0 and HHblits-omitted model is insignificant (*P* < 0.15). We also analyzed the performance of Model 0 with the removal of the LSTM layers. The performance between the modified and original Model 0 is comparable for AUC_ROC_, but is significantly worse in terms of AUC_PR_ and MCC, indicating that the combination of LSTM and IncReSeNet layers adds significant performance gains to the ensemble as a whole.

### Improved disorder prediction over existing techniques

**Figure 2:**
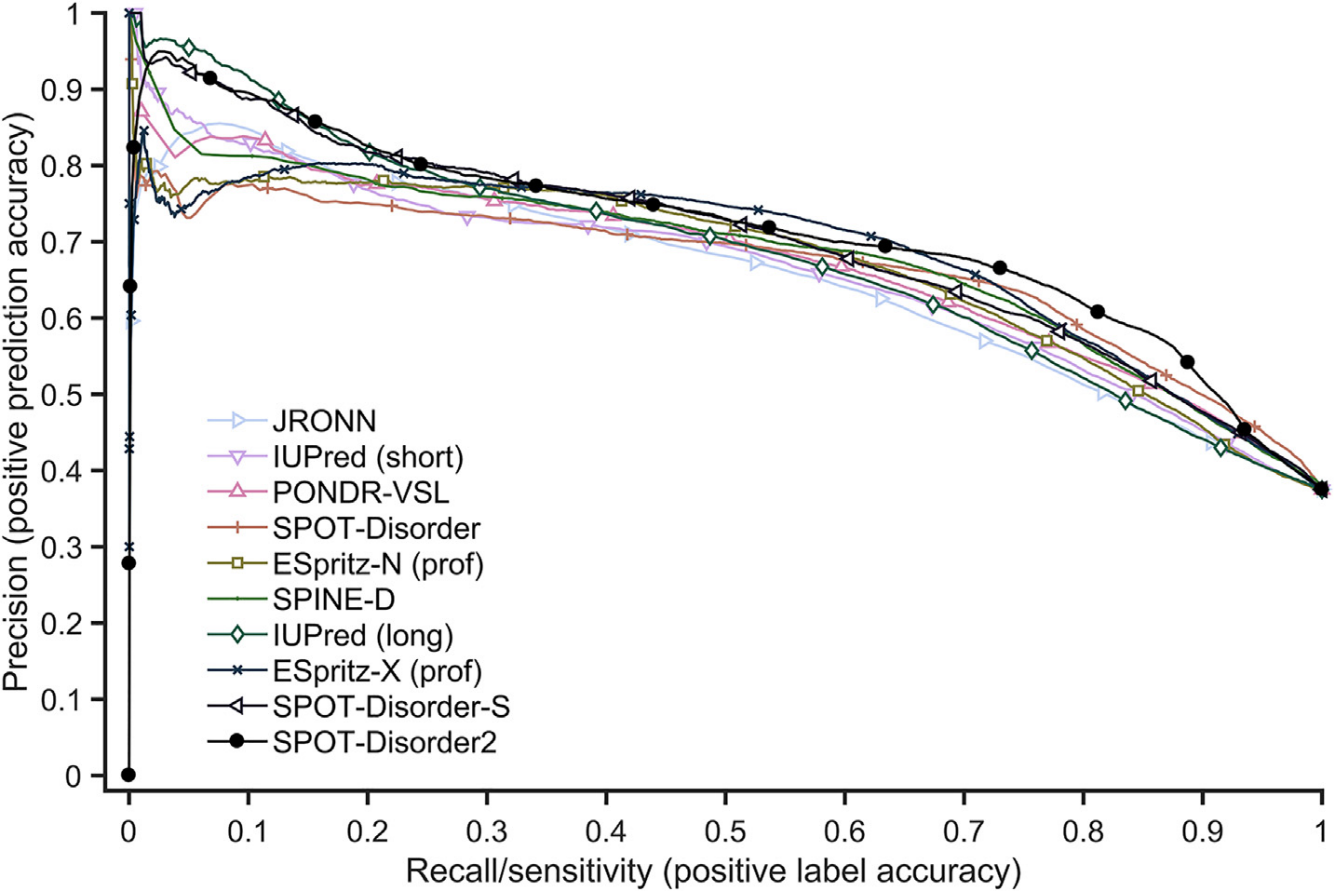
**Precision–recall curves of the top 10 predictors for the DisProt228 dataset** The precision–recall curves were plotted by varying the threshold for defining disordered residues. ESpritz-N (prof) and ESpritz-X (prof) indicate profile-based ESpritz methods trained based on structural information obtained from PDB as determined by NMR or X-ray crystallography, respectively. SPOT-Disorder-S stands for SPOT-Disorder-Single.

We further compared the prediction performance of SPOT-Disorder2 with that of 26 other predictors using the newest annotated proteins in DisProt (DisProt228) [44]. The results of all methods except SPOT-Disorder, NetSurfP-2.0, and SPOT-Disorder2 were obtained by Necci et al. [44]. JRONN [67], IUPred optimized for short and long disorder [IUpred (short)] and [IUpred (long)] [15], and PONDR-VSL [14] are not discussed here because of lower performance except the second-best shown below. However, predictions for two proteins were missing from these data, so the comparisons in this section are based on a 226-protein subset of DisProt228. As shown in Table 4, SPOT-Disorder2 improves over the second-best ESpritz-X (prof) by 2% in AUC_ROC_, 4% in AUC_PR_, 5% in MCC, and 5% in Sw. The precision–recall curves of the top 10 predictors according to AUC_PR_ are shown in Figure 2. The curve for SPOT-Disorder2 is above all other curves at all sensitivity values tested, except that its performance is slightly worse than that of IUpred (long) at sensitivity <0.15, or ESpritz-X (prof) at sensitivity between 0.4 and 0.6. It should be noted that ESpritz-X (prof) has very poor precision at extremely low sensitivity (or near the highest possible threshold that separate disordered residues from ordered residues), suggesting that false positives exist even for the highest confidence scores when using ESpritz-X (prof). The difference between AUC_ROC_ from SPOT-Disorder2 and that from ESpritz-X (prof) is statistically significant (*P* < 1 × 10^−5^, bivariate statistical test).

**Table 4:** **Performance of various disorder prediction methods on a 226-chains subset of the DisProt228 dataset**

*Note*: Performance of NetSurfP-2.0, SPOT-Disorder, SPOT-Disorder-S, and SPOT-Disorder2 was obtained from this work, whereas performance of other methods was reported previously [44]. Two proteins were missing in the DisProt228 dataset [44], so the results here are calculated from the remaining 226 chains. MCC and Sw values for SPOT-Disorder2 were obtained using the disorder probability thresholds that maximize MCC and Sw on the Validation dataset. AUC_PR_ labeled with # is unreliable because the sensitivity (recall) does not cover the whole range from 0 to 1 for the respective methods. seq and prof indicate single sequence-based and sequence profile-based, respectively. See above-mentioned references [64], [65], [66], [68], [69], [70] for further information.

DisProt provides experimental evidence for the labels of about 50% of the residues in the dataset [44]. The remaining ‘undefined’ residues are labeled by DisProt as ordered by default, which would likely introduce some mis-classification of disordered residues. The PR curve is particularly affected by label error due to the increased susceptibility to false positive predictions. We speculate that this label error may account for the (0,0) point of the SPOT-Disorder2 PR curve as well as the poor performance of several methods at low precision (ESpritz-X, MetaDisorder-md2, *etc*.). For example, the first 33 residues for actin-related protein 8 (UniProt: Q9H981; DisProt: DP00873) are amongst the highest confidence disorder prediction hits by SPOT-Disorder2. Despite being labeled as ordered by DisProt, there is no experimental evidence to support this labeling as these residues are missing from the solved X-ray structure [45], [53]. However, we opt not to remove ambiguous residues from the dataset as they do not change the performance ranking of the methods compared. Furthermore, SPOT-Disorder2 shows consistent improvement in terms of other metrics that are more robust to potential label noise, as well as in other datasets where undefined residues have been excluded (*e.g.*, MobiDB).

**Figure 3:**
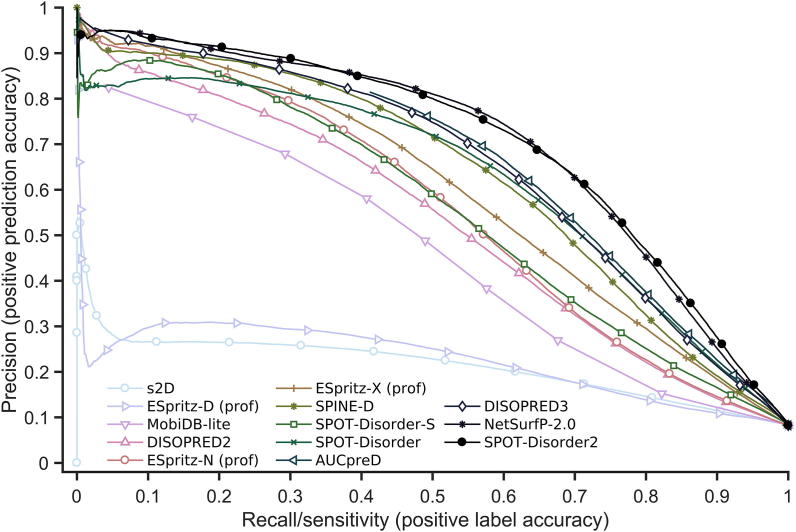
**Precision–recall curves of 13 predictors for the Mobi4730 dataset** The methods compared are s2D, ESpritz-D (prof), MobiDB-lite, DISOPRED2, ESpritz-N (prof), ESpritz-X (prof), SPINE-D, SPOT-Disorder-S, SPOT-Disorder, AUCpreD, DISOPRED3, NetSurfP-2.0, and SPOT-Disorder2.

We further employed other independent test datasets to compare our methods with other top performing methods for DisProt228 that are available to us as either a local implementation or online server. The performance of other methods for Mobi4730 after excluding training datasets is shown in Table 5. ESpritz-X (prof), the second-best predictor for DisProt228, performs significantly worse than SPOT-Disorder2 for Mobi4730, with a 19% difference in AUC_PR_ and 47% difference in MCC. The second best for Mobi4730 is NetSurfP-2.0. SPOT-Disorder2 achieved a 1% increase in AUC_PR_ and a 2.5% increase in MCC over NetSurfP-2.0 for Mobil4730, while the corresponding improvements are 17% in AUC_PR_ and 18% in MCC for DisProt228, respectively. PR curves for all the methods tested are shown in Figure 3. SPOT-Disorder2 has only a slight edge over NetSurfP-2.0, but both are significantly better than other methods examined. It is noted that AUCpreD is optimized for AUC_ROC_, but performs poorly in terms of AUC_PR_. Low AUC_PR_ values result from the inability of methods, such as AUCpreD and MobiDB-lite, to resolve high-confidence true and false positives for this dataset. For example, the PR curve of AUCpreD ends at roughly a sensitivity of 0.4 and a precision of 0.83 because AUCpreD predicts a high number of false positives even when the predicted disorder probability is 1. Calculating AUC without complete coverage of sensitivity from 0 to 1 makes the AUC_PR_ value somewhat arbitrary. To stress the inapplicability of this metric to AUCpreD (and others), we have included a note in Table 4, Table 5, S3, and S4 for the predictors whose sensitivity values do not reach close to 0 and therefore having significantly disadvantaged AUC_PR_ scores. Nevertheless, the AUC_ROC_ of SPOT-Disorder2 is still significantly better than that of the nearest competitor, NetSurfP-2.0 (*P <* 1 × 10^−7^, bivariate statistical test).

**Table 5:** **Performance of various disorder prediction methods on the Mobi4730 dataset**

**Method**	**AUC_ROC_**	**AUC_PR_**	**MCC**	**Sw**
s2D	0.761	0.215	0.234	0.409
ESpritz-D (prof)	0.762	0.226	0.274	0.366
MobiDB-lite	0.811	0.434^#^	0.45	0.449
DISOPRED2	0.859	0.536	0.394	0.577
ESpritz-N (prof)	0.864	0.567	0.299	0.524
SPOT-Disorder-S	0.878	0.567	0.51	0.394
ESpritz-X (prof)	0.893	0.608	0.439	0.635
SPINE-D	0.904	0.644	0.469	0.661
DISOPRED3	0.912	0.641	0.601	0.531
SPOT-Disorder	0.913	0.638	0.595	0.562
AUCpreD	0.917	0.297^#^	0.603	0.611
NetSurfP-2.0	0.926	0.716	0.632	0.511
SPOT-Disorder2	0.933	0.723	0.648	0.715

*Note*: MCC and Sw values for SPOT-Disorder2 were obtained using the disorder probability thresholds that maximize MCC and Sw on the Validation dataset. AUC_PR_ labeled with # is unreliable because the sensitivity (recall) does not cover the whole range from 0 to 1 for the respective methods.

**Figure 4:**
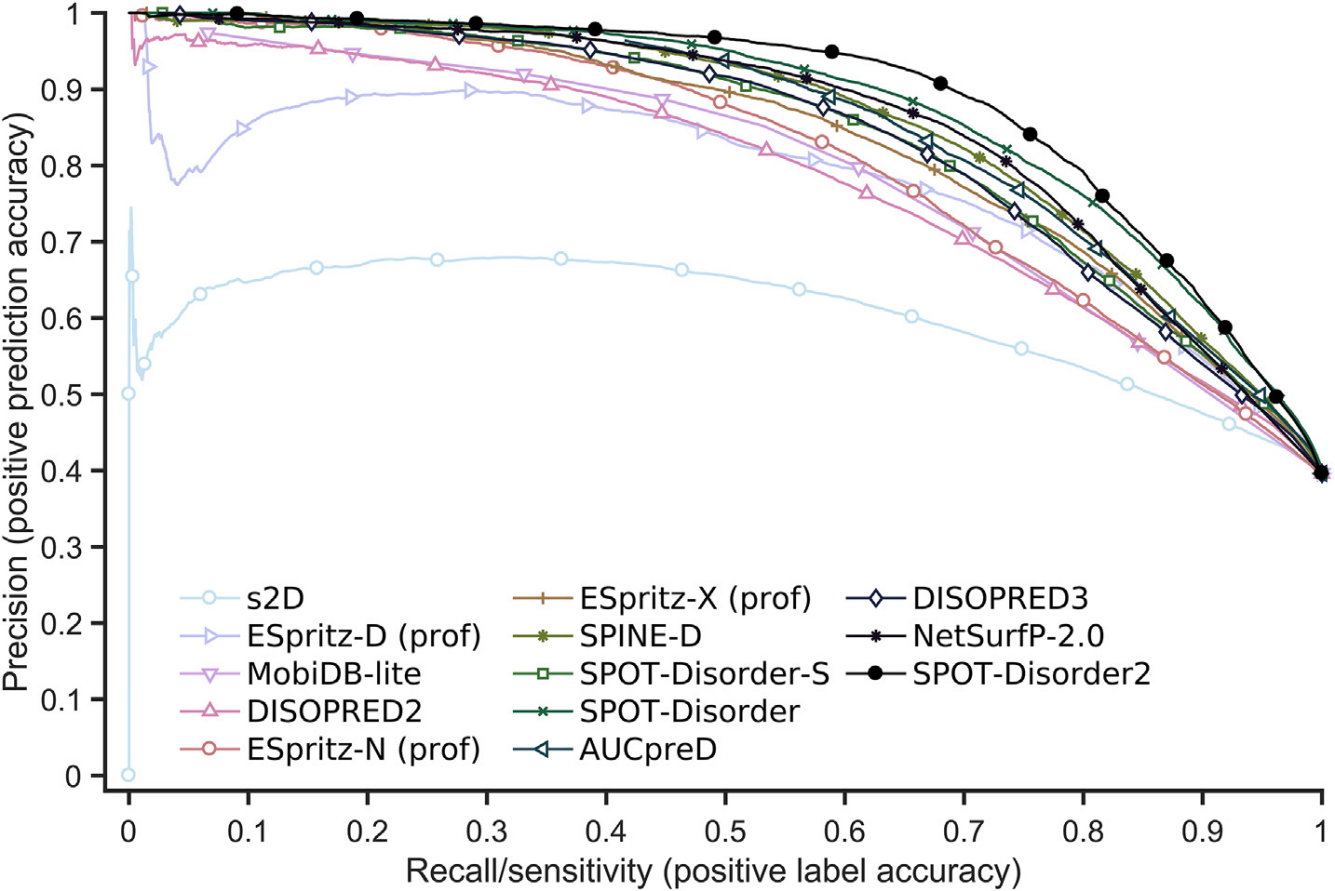
**Precision–recall curves of 13 predictors for the SL250 dataset**

To further demonstrate the stability of the performance of SPOT-Disorder2, we repeated the performance comparison of the aforementioned methods for the SL250 dataset. As shown in Table S3 and Figure 4, SPOT-Disorder2 continues to be the best performer with SPOT-Disorder being the second best. The PR curve of SPOT-Disorder2 is clearly above the curves of all other predictors for this dataset, including SPOT-Disorder and the two second-best methods for the two datasets tested previously, NetSurfP-2.0 and ESpritz-X (prof). The difference in AUC_ROC_ is significant between SPOT-Disorder2 and the nearest predictor AUCpreD (*P* < 1 × 10^−7^, bivariate statistical test), as well as between the SPOT-Disorder2 and SPOT-Disorder (*P* < 1 × 10^−3^, bivariate statistical test) according to a bivariate statistical test.

### Application of SPOT-Disorder2 to long proteins

Analysis on the UniProtKB/Swiss-Prot database (as of Dec 2018) [54] has shown that more than 91% of proteins consist of <700 AA residues, indicating that SPOT-Disorder2 is applicable to the vast majority of available sequences. However, it is also important to see how SPOT-Disorder2 performs for longer proteins representative of the remaining 9% that are not covered. Note that the size of 700 AA residues is not a hard limit in the software, but the size which was found to maximize the memory usage of GPU on our workstation.

The size limitation is mainly due to the use of SPOT-1D in the input of SPOT-Disorder2 input, which relies on the contact map prediction tool SPOT-Contact [25]. The computational memory necessary for using SPOT-1D with extremely long sequences becomes far too high for a typical user’s workstation. To test the utility of SPOT-Disorder2 for long proteins, we replaced SPOT-1D by the secondary structure prediction tool SPIDER3 [55]. We generated the disorder profiles of SPOT-Disorder2 for 31 proteins that were initially omitted from the DisProt complement set from Necci et al. [44] in DisProt228, using the outputs of the secondary structure prediction tool SPIDER3 [55] in place of SPOT-1D (one protein consisting of >18,000 AA residues is still omitted). As SPIDER3 does not predict for 8-state secondary structure, we merely assign the 3-state probability predictions of SPIDER3 to the C, H, and E states for the 8-state predictions (and 0 for the S, T, I, G, and B states).

We compared the modified SPOT-Disorder2 to other methods for 31 large proteins (consisting of >700 AA residues) that were initially omitted from the DisProt complement set from Necci et al. [44] in DisProt228. Table S4 shows that s2D is the top predictor for long proteins although it was the worst predictor for Mobi4730 and SL250, indicating that the disordered residues in the large-protein dataset tend to be in a coil state. However, the MCC of s2D is poor. SPOT-Disorder2 drops in the rankings, as is expected due to the learned distribution of the secondary structure inputs changing from SPOT-1D to SPIDER3, as well as losing the information from the 8-state secondary structure. The higher performance of SPOT-Disorder-S (highest MCC of 0.457) for this set of 31 proteins with >700 AA residues might be explained by the fact that profile-based models are not well-trained for large proteins consisting of >700 AA residues. A single-sequence-based method, on the other hand, is less dependent on sequence length. This is also echoed in the performance of single sequence-based ESpritz-D (seq) against the sequence profile-based ESpritz-D (prof) method (MCC of 0.382 *vs*. 0.228, respectively). Nevertheless, SPOT-Disorder2 is still one of the higher-ranking predictors, indicating that it is useful for long protein chains as well.

### SPOT-Disorder2 is less accurate for the proteins with few sequence homologies

**Figure 5:**
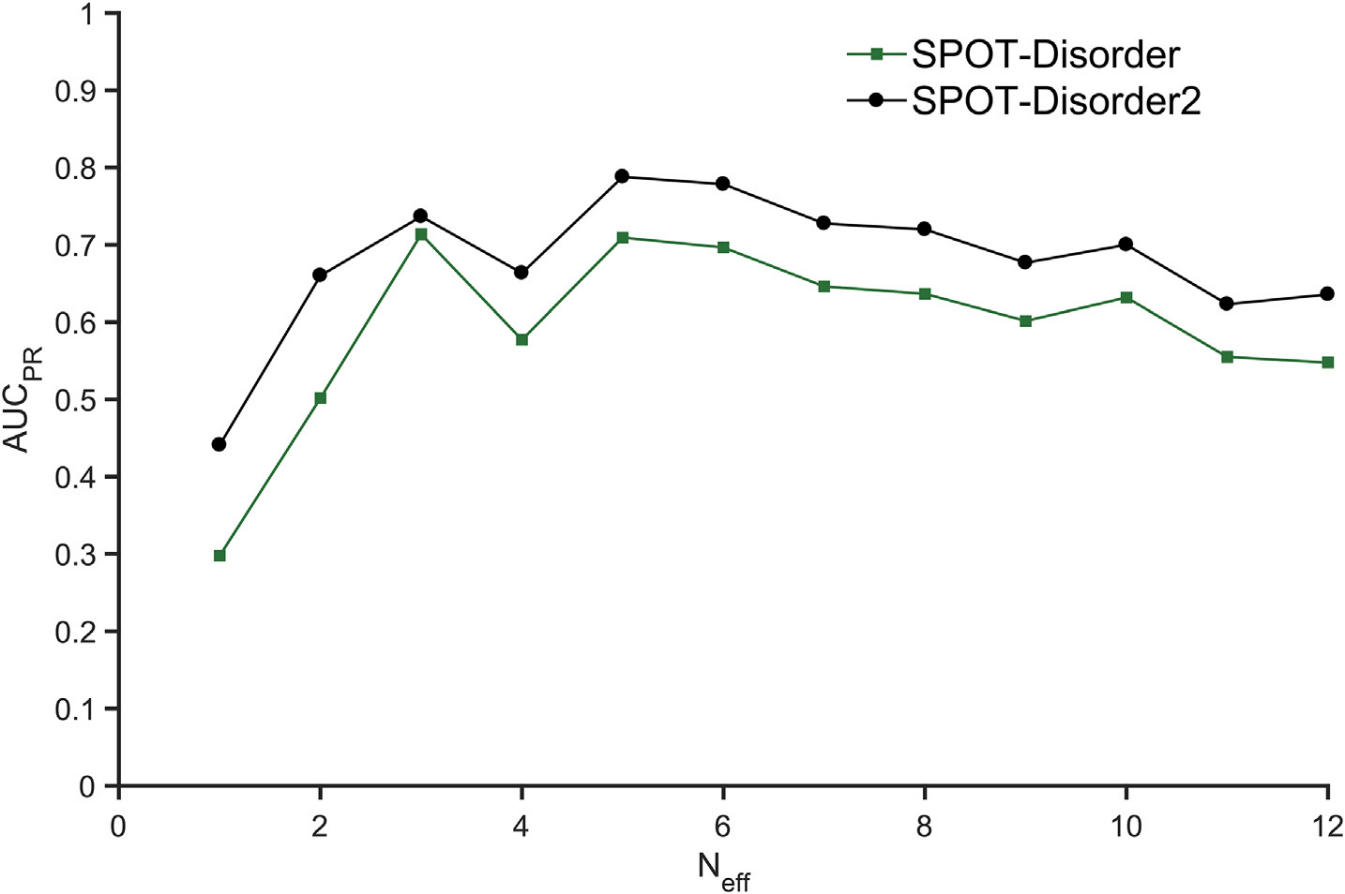
**AUC_PR_ for proteins with different Neff values generated from HHblits** AUC_PR_, area under precision-recall curve; Neff, number of effective homologous sequences of a given protein.

Robust performance of SPOT-Disorder2 across different datasets can be attributed to the evolutionary information derived from multiple sequence alignments in PSI-BLAST and HHBlits. To examine the contribution of evolutionary information, we evaluated the performance of disorder prediction according to AUC_PR_ as a function of Neff. The larger Mobi9414 set is used, so that we have sufficient statistics for different values of Neff. As Figure 5 shows, SPOT-Disorder2 performs more accurately for proteins with Neff > 5, below which there is a sharp decline in performance. However, there is a drop for proteins with Neff > 6. Significant homology between sequences seems to introduce noise into our prediction of disordered regions, indicating that these regions might not be conserved like structured regions. Another possible cause is the sensitivity of disorder prediction to false positives in the homolog search. More studies are needed to isolate the cause of this pattern. Nevertheless, SPOT-Disorder2 makes significant improvement over SPOT-Disorder at all Neff values even for sequences with little evolution information (Neff ≈ 1). This suggests that improvement is possible even at the single sequence level when several advanced machine learning techniques are integrated for consensus prediction.

### Application of SPOT-Disorder2 to prediction of binding regions in disordered regions

Some intrinsically disordered regions can fold when interacting with other molecules including proteins, while others are structureless under any circumstances. Separating these foldable and non-foldable disordered regions is important for identification of functional regions, or MoRFs. Previously, we have proposed that foldable disordered regions are in a semi-disordered state with predicted disordered probabilities ranging from fully disordered [p(D) = 1] to fully structured [(p(D) = 0] [56]. We tested this hypothesis using the output predictions from SPOT-Disorder2.

We have downloaded the Test and Test2012 datasets from the MoRFpred server [57] (http://biomine.cs.vcu.edu/servers/MoRFpred/) for validation and independent testing, respectively. We removed redundant sequences between the Test2012 and Test datasets at 25% sequence similarity using BLASTClust and the proteins with >700 AA residues. As a result, 220 and 22 chains from Test2012 and Test datasets were retained for further analysis, respectively. The smoothing window size, along with the upper and lower thresholds, are optimized on the Test dataset. For comparison with other models, besides the web servers of MoRFpred, fMoRFpred, and DisoRDPbind [58], [59] (http://biomine.cs.vcu.edu/#webservers), we also used ANCHOR2 [60] (https://iupred2a.elte.hu/), MoRFchibi [61] (https://gsponerlab.msl.ubc.ca/software/morf_chibi/), and the local version of MoRFPred-plus [62] (https://github.com/roneshsharma/MoRFpred-plus). In addition, we compared our results to the prediction by DISOPRED3 [49].

The performance of all predictors on the subset (220 chains) of the Test2012 dataset is shown in Table S5. With only three parameters trained for the Test dataset, SPOT-Disorder2 outperforms the second best MoRFPred-plus for MoRF prediction of the Test2012 dataset in terms of MCC (0.155 by SPOT-Disorder2 compared to 0.143 by MoRFPred-plus). Unlike SPOT-Disorder2, all other methods were specifically trained for MoRF regions. However, the performance of all methods is low, with MCC < 0.2. More data might be needed to further improve these methods for predicting binding residues in disordered regions.

## Conclusion

In this paper, we have introduced a new method for predicting protein intrinsic disorder by taking advantage of recent progress in image recognition. With regard to the neural network architecture, we implemented two recent developments for an extension on residual convolutional neural networks, *i.e.*, multiple inception-style pathways [30] and signal Squeeze-and-Excitation [29]. We have also updated our feature set from our previous work [19] to include the latest state-of-the-art predictions for protein secondary structure from SPOT-1D [27]. Finally, the use of an ensemble of these methods has been again demonstrated effective in increasing accuracy through the removal of spurious false predictions. These enhancements over our previous and other disorder predictors enables SPOT-Disorder2 to achieve more robust and higher performance across different datasets with varied disorder to order ratios. Consequently, SPOT-Disorder2 achieves the best performance over all metrics analyzed among the predictors tested.

MMSeqs2 [63] is considered in this study due to its speedup in generating profiles over PSI-BLAST. However, MMSeqs2 produces a less accurate prediction if its profiles are directly used to replace the profiles from PSI-BLAST, partially because SPOT-Disorder2 is trained on PSI-BLAST profiles. We hope to train a model for disorder prediction based on MMSeqs2 profiles in a future work.

## Availability

SPOT-Disorder2 is available at https://sparks-lab.org/server/spot-disorder2/ as a server and downloadable package for local implementation.

## Authors’ contributions

JH, KKP, and YZ conceived and designed the experiments. TL prepared the data. JH conducted the experiments. JH, TL, and YZ analyzed the data and wrote the paper. All authors read, revised, and approved the final manuscript.

## Competing interests

The authors declare no conflicts of interest.
