# Supplementary material for: SPOT-Disorder2: Improved Protein Intrinsic Disorder Prediction by Ensembled Deep Learning
Source: Genomics Proteomics Bioinformatics. 2020 Mar 13;17(6):645–56. doi: 10.1016/j.gpb.2019.01.004 (PMC7212484; doi:10.1016/j.gpb.2019.01.004)
Supplement: Supplementary Table S1 [file mmc1.docx]

| **Table S1 Performance of the individual models from Table 1 on the Mobi9414 dataset alongside the performance of the final ensemble** | | | | |
| --- | --- | --- | --- | --- |
| **Model** | **AUC_ROC_** | **AUC_PR_** | **MCC** | **Sw** |
| Model 0 | 0.934 | 0.681 | 0.621 | 0.732 |
| Model 1 | 0.928 | 0.685 | 0.628 | 0.713 |
| Model 2 | 0.941 | 0.697 | 0.633 | 0.743 |
| Model 3 | 0.936 | 0.683 | 0.624 | 0.72 |
| Model 4 | 0.936 | 0.679 | 0.617 | 0.734 |
| SPOT-Disorder2 | 0.943 | 0.71 | 0.642 | 0.744 |
| *Note*: AUC_ROC_, area under the receiver operating characteristic curve; AUC_PR_, area under the precision–recall curve; MCC, Matthew’s correlation coefficient; Sw, weighted score. MCC and Sw values were obtained using the thresholds that maximize MCC and Sw on the Validation dataset. | | | | |
