# Supplementary material for: SPOT-Disorder2: Improved Protein Intrinsic Disorder Prediction by Ensembled Deep Learning
Source: Genomics Proteomics Bioinformatics. 2020 Mar 13;17(6):645–56. doi: 10.1016/j.gpb.2019.01.004 (PMC7212484; doi:10.1016/j.gpb.2019.01.004)
Supplement: Supplementary Table S2 [file mmc2.docx]

| **Table S2 Performance of Model 0 from Table 1 on the Mobi9414 dataset with the removal of input feature groups, alongside a non-LSTM Model 0 variant** | | | | |
| --- | --- | --- | --- | --- |
| **Model** | **AUC_ROC_** | **AUC_PR_** | **MCC** | **Sw** |
| Model 0 | 0.9343 | 0.681 | 0.621 | 0.732 |
| PSSM-omit | 0.9181 | 0.227 | 0.61 | 0.698 |
| HHblits-omit | 0.935 | 0.687 | 0.626 | 0.734 |
| SPOT-1D-omit | 0.92 | 0.623 | 0.576 | 0.69 |
| IncReSeNet only | 0.9301 | 0.659 | 0.601 | 0.723 |
| *Note*: MCC and Sw are obtained using the thresholds that maximize MCC and Sw on the Validation dataset. | | | | |
