# Supplementary material for: SPOT-Disorder2: Improved Protein Intrinsic Disorder Prediction by Ensembled Deep Learning
Source: Genomics Proteomics Bioinformatics. 2020 Mar 13;17(6):645–56. doi: 10.1016/j.gpb.2019.01.004 (PMC7212484; doi:10.1016/j.gpb.2019.01.004)
Supplement: Supplementary Table S3 [file mmc3.docx]

| **Table S3 Performance of various methods on the SL250 dataset** | | | | |
| --- | --- | --- | --- | --- |
| **Model** | **AUC_ROC_** | **AUC_PR_** | **MCC** | **Sw** |
| s2D | 0.737 | 0.604 | 0.36 | 0.368 |
| MobiDB-lite | 0.818 | 0.730^#^ | 0.534 | 0.471 |
| DISOPRED2 | 0.825 | 0.786 | 0.508 | 0.504 |
| ESpritz-N (prof) | 0.833 | 0.812 | 0.454 | 0.462 |
| ESpritz-D (prof) | 0.843 | 0.776 | 0.555 | 0.544 |
| DISOPRED3 | 0.857 | 0.796 | 0.596 | 0.56 |
| ESpritz-X (prof) | 0.859 | 0.835 | 0.566 | 0.57 |
| SPOT-Disorder-S | 0.862 | 0.84 | 0.542 | 0.442 |
| NetSurfP-2.0 | 0.869 | 0.834 | 0.572 | 0.477 |
| AUCpreD | 0.869 | 0.445^#^ | 0.605 | 0.547 |
| SPINE-D | 0.875 | 0.854 | 0.599 | 0.602 |
| SPOT-Disorder | 0.893 | 0.875 | 0.629 | 0.567 |
| SPOT-Disorder2 | 0.901 | 0.889 | 0.679 | 0.625 |
| *Note*: MCC and Sw values for SPOT-Disorder2 were obtained using the disorder probability thresholds that maximize MCC and Sw on the Validation dataset. AUC_PR_ labelled with # is unreliable because the sensitivity (recall) does not cover the whole range from 0 to 1 for the respective methods. | | | | |
