# Supplementary material for: SPOT-Disorder2: Improved Protein Intrinsic Disorder Prediction by Ensembled Deep Learning
Source: Genomics Proteomics Bioinformatics. 2020 Mar 13;17(6):645–56. doi: 10.1016/j.gpb.2019.01.004 (PMC7212484; doi:10.1016/j.gpb.2019.01.004)
Supplement: Supplementary Table S4 [file mmc4.docx]

| **Table S4 Performance of various methods on 31 proteins containing > 700 AA residues from the DisProt Complement dataset** | | | | |
| --- | --- | --- | --- | --- |
| **Method** | **AUC_ROC_** | **AUC_PR_** | **MCC** | **Sw** |
| GlobPlot | 0.051 | 0.218 | 0.15 | 0.085 |
| MetaDisorder-md | 0.551 | 0.163^#^ | 0.355 | 0.48 |
| MetaDisorder-3D | 0.559 | 0.118^#^ | 0.057 | 0.08 |
| DisEMBL (Hot-Loops) | 0.623 | 0.198 | 0.096 | 0.126 |
| MetaDisorder | 0.631 | 0.025^#^ | 0.349 | 0.472 |
| DisEMBL (465) | 0.648 | 0.222 | 0.126 | 0.128 |
| MetaDisorder-md2 | 0.683 | 0.234 | 0.324 | 0.448 |
| JRONN | 0.729 | 0.278 | 0.282 | 0.39 |
| NetSurfP-2.0 | 0.747 | 0.269 | 0.247 | 0.283 |
| IUPred (short) | 0.752 | 0.279 | 0.288 | 0.336 |
| Espritz-X (seq) | 0.755 | 0.31 | 0.319 | 0.373 |
| Espritz-N (seq) | 0.76 | 0.323 | 0.326 | 0.439 |
| AUCpreD | 0.767 | 0.127^#^ | 0.42 | 0.519 |
| PONDR-VSL | 0.768 | 0.355 | 0.277 | 0.387 |
| IUPred (long) | 0.768 | 0.295 | 0.367 | 0.47 |
| MobiDB-lite | 0.772 | 0.324^#^ | 0.319 | 0.327 |
| ESpritz-D (prof) | 0.781 | 0.356 | 0.228 | 0.179 |
| ESpritz-N (prof) | 0.784 | 0.36 | 0.348 | 0.47 |
| ESpritz-D (seq) | 0.788 | 0.389 | 0.382 | 0.321 |
| ESpritz-X (prof) | 0.795 | 0.361 | 0.413 | 0.523 |
| DISOPRED | 0.796 | 0.337^#^ | 0.359 | 0.485 |
| MFDp | 0.8 | 0.406 | 0.347 | 0.475 |
| MFDp2 | 0.807 | 0.459 | 0.393 | 0.513 |
| SPINE-D | 0.808 | 0.463 | 0.332 | 0.459 |
| SPOT-Disorder2 | 0.821 | 0.435 | 0.404 | 0.532 |
| SPOT-Disorder-S | 0.821 | 0.496 | 0.457 | 0.541 |
| SPOT-Disorder | 0.825 | 0.467 | 0.428 | 0.537 |
| s2D | 0.839 | 0.544 | 0.281 | 0.389 |
| *Note*: Performance of NetSurfP-2.0, SPOT-Disorder, SPOT-Disorder-S, and SPOT-Disorder2 was obtained from this work, whereas performance of other methods was reported previously [44]. MCC and Sw values for SPOT-Disorder2 were obtained using the disorder probability thresholds that maximize MCC and Sw on the Validation dataset. AUC_PR_ labelled with # is unreliable because the sensitivity (recall) does not cover the whole range from 0 to 1 for the respective methods. seq and prof indicate single sequence-based and sequence profile-based, respectively. | | | | |
