# Supplementary material for: SPOT-Disorder2: Improved Protein Intrinsic Disorder Prediction by Ensembled Deep Learning
Source: Genomics Proteomics Bioinformatics. 2020 Mar 13;17(6):645–56. doi: 10.1016/j.gpb.2019.01.004 (PMC7212484; doi:10.1016/j.gpb.2019.01.004)
Supplement: Supplementary Table S5 [file mmc5.docx]

| **Table S5 Performance of various methods on the Test2012 dataset** | | | | |
| --- | --- | --- | --- | --- |
| **Method** | **MCC** | **Sensitivity** | **Specificity** | **Precision** |
| DISOPRED3 | 0.024 | 11.48 | 92.14 | 4.98 |
| fMoRFpred | 0.083 | 9.26 | 97.77 | 12.95 |
| DisoRDPbind | 0.101 | 20 | 93.77 | 10.33 |
| MoRFpred | 0.125 | 23.7 | 93.67 | 11.83 |
| MoRFPred-plus | 0.143 | 60 | 74.59 | 7.81 |
| ANCHOR2 | 0.079 | 45.93 | 73.32 | 5.81 |
| MoRFchibi | 0.115 | 13.33 | 97.4 | 15.52 |
| SPOT-Disorder2 | 0.155 | 64.07 | 73.77 | 8.05 |
| *Note*: Dual thresholds for SPOT-Disorder2 are set to 0.125 and 0.867. Window size (*w_L_*) is 12 from the target residue. | | | | |
